# Supplementary material for: Efficacy of Nrf2 activation in a proteinuric Alport syndrome mouse model
Source: Life Sci Alliance. 2025 Jun 17;8(8):e202503330. doi: 10.26508/lsa.202503330 (PMC12174586; doi:10.26508/lsa.202503330)
Supplement: Supplementary file 1 [file LSA-2025-03330_SdataFS8.pdf]

Source Data for Figure S8A

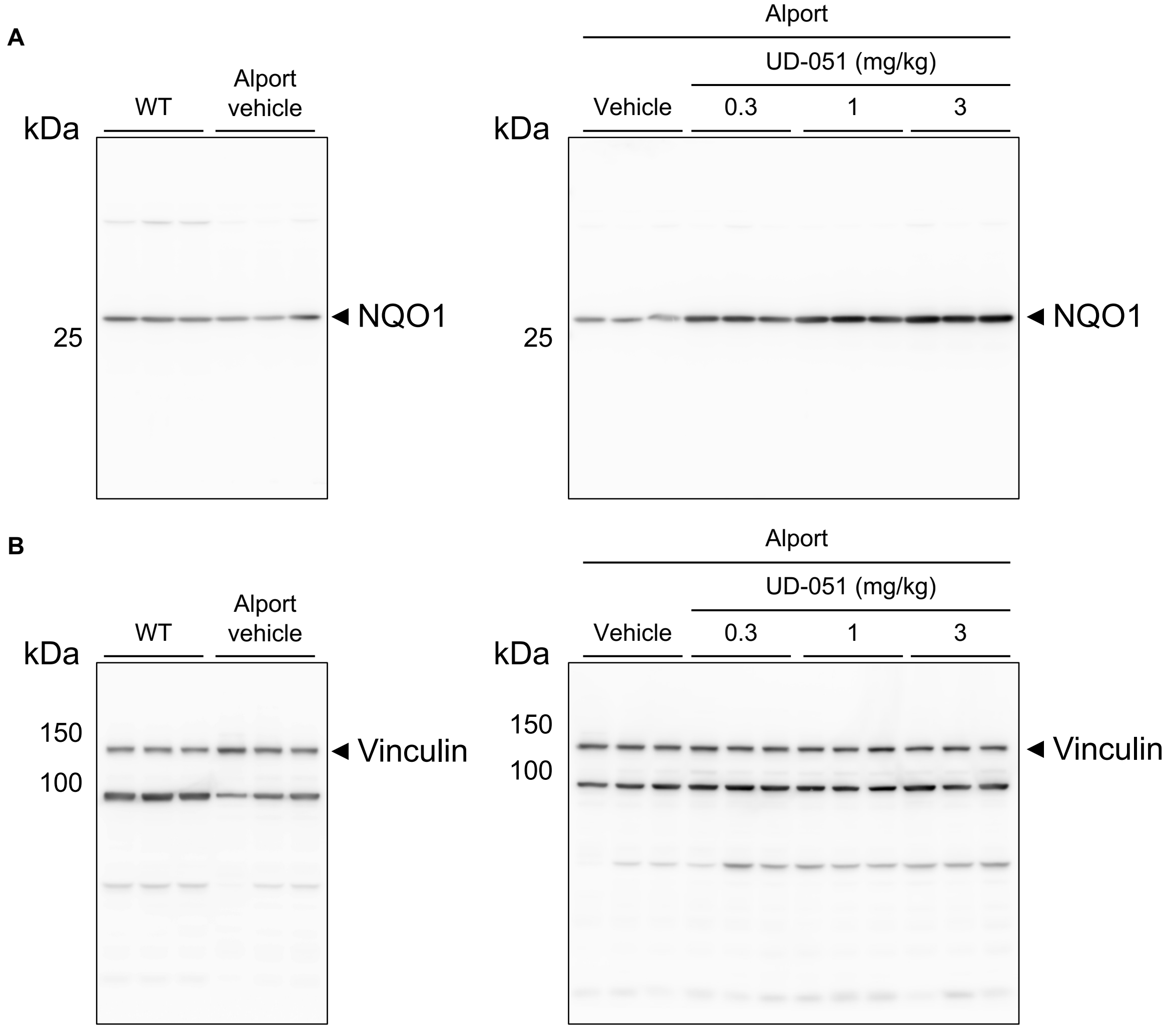

Full length blots for Figure S8A

The full-length blots for Figure S8A with the indicated antibodies. Vinculin was used as a loading control. Samples were derived from the same experiment, and gels/blots were processed in parallel.
