# Supplementary material for: Efficacy of Nrf2 activation in a proteinuric Alport syndrome mouse model
Source: Life Sci Alliance. 2025 Jun 17;8(8):e202503330. doi: 10.26508/lsa.202503330 (PMC12174586; doi:10.26508/lsa.202503330)
Supplement: Supplementary file 2 [file LSA-2025-03330_TableS1.docx]

**Table S1.** **Primers used in quantitative RT-PCR.**

h=Human and m=Mouse

| Gene | Sense | Antisense |
| --- | --- | --- |
| *hNrf2* | 5’-ATTGTGCCTTCAGCGTGCTT-3’ | 5’-TCCGCTGCCATCAGTCAGTC-3’ |
| *hNqo1* | 5’-CAGCCAATCAGCGTTCGGTA-3’ | 5’-CTTCATGGCGTAGTTGAATGATGTC-3’ |
| *hActb* | 5’-CATCCGTAAAGACCTCTATGCCAAC-3’ | 5’-GGAGCCACCGATCCACA-3’ |
| *mIl-1b* | 5’-TCCAGGATGAGGACATGAGCAC-3’ | 5’-GAACGTCACACACCAGCAGGTTA-3’ |
| *mTnf-α* | 5’-TATGGCCCAGACCCTCACA-3’ | 5’-GGAGTAGACAAGGTACAACCCATC-3’ |
| *mCxcl10* | 5’-ATCATCCCTGCGAGCCTATCC-3’ | 5’-TGTCCATCCATCGCAGCAC-3’ |
| *mTgf-β* | 5’-TACGGCAGTGGCTGAACCAA-3’ | 5’-CGGTTCATGTCATGGATGGTG-3’ |
| *mCol1a1* | 5’-GACATGTTCAGCTTTGTGGACCTC-3’ | 5’-GGGACCCTTAGGCCATTGTGTA-3’ |
| *mKim-1* | 5’-GGAAGTAAAGGGGGTAGTGGG-3’ | 5’-AAGCAGAAGATGGGCATTGC-3’ |
| *mLcn2* | 5’-GGAACGTTTCACCCGCTTTG-3’ | 5’-GTCTCTGCGCATCCCAGTCA-3’ |
| *mIl-6* | 5’-CCACTTCACAAGTCGGAGGCTTA-3’ | 5’-CCAGTTTGGTAGCATCCATCATTTC-3’ |
| *mMmp2* | 5’-AGAACTTCCGATTATCCCATGATGA-3’ | 5’-TGACAGGTCCCAGTGTTGGTG-3’ |
| *mMmp14* | 5’-CTCCACAAAGATCTGCCTCTATCCA-3’ | 5’-CGGGTTTGCCAAGGGTTTC-3’ |
| *mLrp2* | 5’-CAGTGGATTGGGTAGCAGGA-3’ | 5’-GCTTGGGGTCAACAACGATA -3’ |
| *mGapdh* | 5’-TGTGTCCGTCGTGGATCTGA-3’ | 5’-TTGCTGTTGAAGTCGCAGGAG-3’ |
